# CTCF Mediates the Cell-Type Specific Spatial Organization of the *Kcnq5* Locus and the Local Gene Regulation

**DOI:** 10.1371/journal.pone.0031416

**Published:** 2012-02-08

**Authors:** Licheng Ren, Yang Wang, Minglei Shi, Xiaoning Wang, Zhong Yang, Zhihu Zhao

**Affiliations:** 1 State Key Laboratory of Bioreactor Engineering, East China University of Science and Technology, Shanghai, China; 2 Beijing Institute of Biotechnology, Fengtai District, Beijing, China; 3 State Key Laboratory of Genetic Engineering and Department of Microbiology and Microbial Engineering, School of Life Sciences, Fudan University, Shanghai, China; The University of Hong Kong, Hong Kong

## Abstract

Chromatin loops play important roles in the dynamic spatial organization of genes in the nucleus. Growing evidence has revealed that the multivalent functional zinc finger protein CCCTC-binding factor (CTCF) is a master regulator of genome spatial organization, and mediates the ubiquitous chromatin loops within the genome. Using circular chromosome conformation capture (4C) methodology, we discovered that CTCF may be a master organizer in mediating the spatial organization of the *kcnq5* gene locus. We characterized the cell-type specific spatial organization of the *kcnq5* gene locus mediated by CTCF in detail using chromosome conformation capture (3C) and 3C-derived techniques. Cohesion also participated in mediating the organization of this locus. RNAi-mediated knockdown of CTCF sharply diminished the interaction frequencies between the chromatin loops of the *kcnq5* gene locus and down-regulated local gene expression. Functional analysis showed that the interacting chromatin loops of the *kcnq5* gene locus can repress the gene expression in a luciferase reporter assay. These interacting chromatin fragments were a series of repressing elements whose contacts were mediated by CTCF. Therefore, these findings suggested that the dynamical spatial organization of the *kcnq5* locus regulates local gene expression.

## Introduction

Eukaryotic chromosomes are intricately folded into sophisticated higher-order structures and packaged in the nucleus [1]. These higher-order packaged chromosomes spatially occupy the so-called ‘chromosome territory’ in the nucleus and play important roles in genome function and the precise regulation of gene expression [2]. Chromatin loops are ubiquitous sub-structural elements of genome spatial organization. The dynamic nature of nuclear spatial organization is highlighted by the mobility of active genes that move from the tightly folded spaces to loop out and relocate, which allows for interaction with other *cis*-regulatory elements [1]–[4]. However, very little is known on how these orchestral components are organized spatially within the nucleus.

CCCTC-binding factor (CTCF) is a highly conserved zinc finger protein that is ubiquitously expressed in metazoa. Emerging evidences has revealed that CTCF is a multivalent factor that has been implicated in diverse cellular processes. CTCF is able to recognize and bind to different DNA motifs through different combinations of its eleven zinc-fingers. There are tens of thousands of CTCF binding sites throughout the genomes of human and mouse. By binding to the insulators or boundary elements, CTCF can demarcate chromatin into independent regulatory regions and block communication between promoters and enhancers to regulate gene expression. As the master organizer of genomic spatial organization, CTCF plays important roles in gene transcriptional activation or repression, genomic imprinting, and X chromosome inactivation [4]–[9].

The development of high-throughput circular chromosome conformation capture (4C) and other related 3C-derived techniques have greatly improved our understanding of the sophisticated organization of the nucleus [10]–[12]. In order to explore the roles of CTCF-mediated chromosomal interactions, a highly conserved CTCF binding site was identified by analyzing the data previously reported [13]. This CTCF binding site was used as 4C bait for the screening of potential interacting partners throughout the genome. The sequence of the 4C bait was matched to the *kcnq5* gene locus. The human KCNQ gene family includes five members that encode K+ channel α-subunits. KCNQ5 is expressed in the brain and skeletal muscle and associates with KCNQ3 to form a potassium channel [14]–[16]. To date, little is known about the regulation of KCNQ5 expression or its gene locus organization. Here, we report that CTCF mediates a series of repressing element interactions that form loops on the *kcnq5* gene locus as a mechanism for regulating local gene expression.

## Results

### Limited 4C screening identified the intra-chromatin interactions within *kcnq5* gene locus

**Figure 1:**
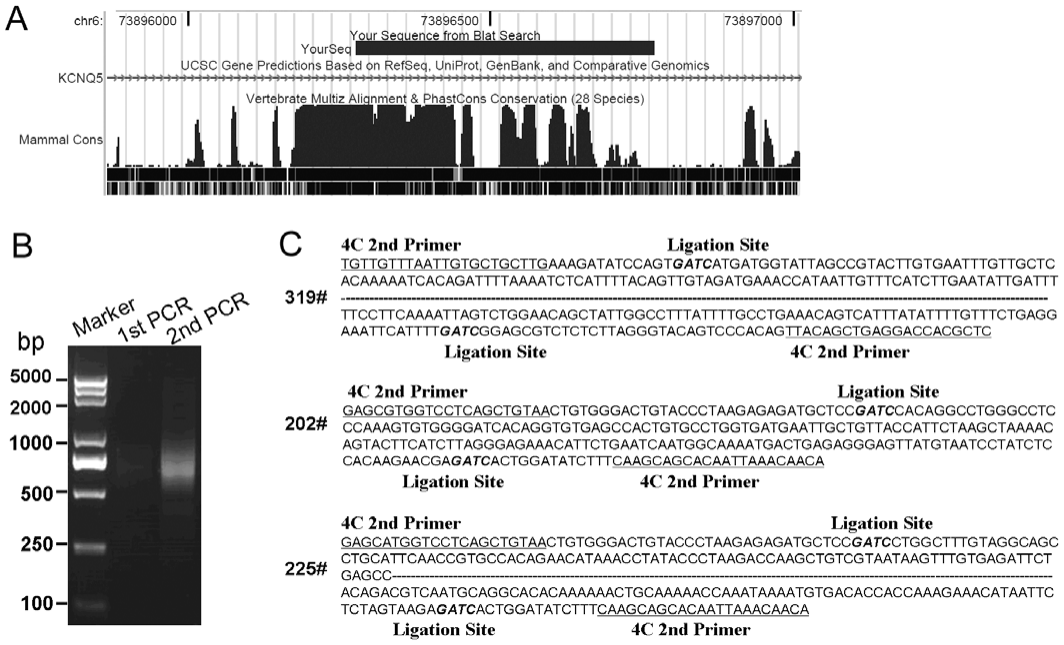
4C assay and limited screening. (A) The bait sequence for 4C assay was a highly conserved DNA fragment of the 28 vertebrate species analyzed using the UCSC Genome Browser. (B) Gel electrophoresis analysis of the product of 4C nested reverse PCR amplification. The results showed the smear-like trails stained with ethidium bromide (EtBr, 0.5 µg/ml). (C) The sequencing results of three interaction partners (No. 319, 202, and 225) were identified by the 4C assay.

The 4C technique is a high-throughput format used to screen the entire genome for unexpected potential interacting partners using a known bait sequence [11], [17]. To investigate the chromosome interaction networks mediated by CTCF, we chose a highly conserved CTCF binding site as the 4C bait that is ubiquitous across different cell lines. Xi *et al.* [13] investigated six diverse human cell types to identify the cell-type specific regulatory elements using DNase-chip. By combining the anti-CTCF ChIP-chip and DNase-chip assays, 26 sites distal to the transcription start site (TSS) were filtered out, on which the ubiquitous DNase I HS (hypersensitive) sites and CTCF binding sites overlapped. We analyzed the 26 ubiquitous CTCF binding sites using the UCSC Genome Browser. The results showed that one highly conserved sequence encompassed a CTCF binding site in several different cell lines, which matched the *kcnq5* gene locus of chromosome 6. We chose this highly conserved CTCF binding sequence as 4C bait and named this *Dpn* II-digested fragment as CT6 (chr6: 73896277–73896771) (Figure 1A).

Pairs of primers for nested reverse PCR of the 4C technique were designed to match bases near the ends of the CT6 fragment bait in order to identify potential interacting partners in MCF-7 cells (Table S1). The nested reverse PCR product was then analyzed by gel electrophoresis and ethidium bromide (EtBr) staining, which exhibited smear-like trails (Figure 1B). The second round PCR product was purified and cloned into T-vectors and subsequently transformed into *E. coli*. The resulting positive clones were screened and further confirmed by sequencing, which showed that most of the resulting sequences matched on chromosome 6. Three of the clones, including No. 225 (chr6: 73308350–73308788), No. 319 (chr6: 73,765,823–73,766,377), and No. 202 (chr6: 73,912,667–73,912,848) were within the same *kcnq5* gene locus (Figure 1C). Analysis of the data sets appearing in ENCONDE on the UCSC Genome Browser showed that these three chromatin fragments did not overlap with the CTCF binding sites that were previously reported. As such, they may be novel CTCF binding sites in MCF-7 cells. The analysis results also showed that there were several CTCF binding sites on the *kcnq5* gene locus, which indicated that CTCF might have a role in the spatial organization of the *kcnq5* gene locus. In order to characterize the role of CTCF in the organization of the *kcnq5* gene locus in more detail, the spatial relationship of the three screened out fragments as well as the other four potential CTCF binding sites on the gene locus was analyzed together.

### Analysis of the spatial organization of the *Kcnq5* locus using the 3C assay

**Figure 2:**
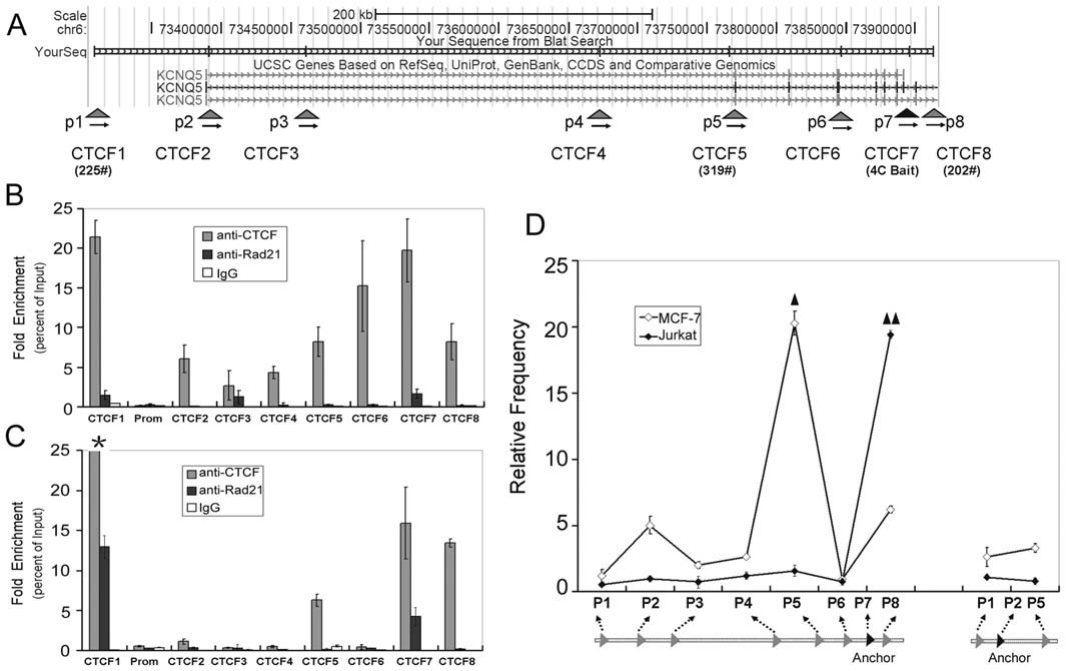
The cell-type specific binding of CTCF and cohesin binding sites and the spatial organization of the *kcnq5* locus. (A) A schematic representation of the chromatin fragments of the *kcnq5* locus for ChIP and 3C assays. The eight *Hind* III-digested chromatin fragments for the 3C assay were aligned using the UCSC Genome Browser, each of which encompassed a potential CTCF binding site. The 3C primers were designed to correspond to the sequences near the downstream sticky ends of the 3C fragments and named p1 to p8 according to their position on the gene locus. The primer pairs for ChIP assay were designed to span each potential CTCF binding sites. Real-Time quantitative PCR analyzed the DNA fragments enriched by anti-CTCF and Rad21 ChIP assays with (B) MCF-7 cells and (C) Jurkat cells (*: the relative enrichment was 82). The independent quantitative PCR amplification efficiency was normalized by the input template. Error bars represent mean ± s.d. (n = 3). (D) 3C quantitative PCR assay detected the interaction frequencies between the chromatin fragments of the *kcnq5* gene locus in MCF-7 and Jurkat cells (the relative interaction frequencies, ▴: 840 and ▴▴: 770). The amplification efficiencies of the independent PCR reactions were normalized with respect to the control template. Error bars represent mean ± s.d. (n = 3).

The 3C assay involves chromatin cross-linking with formaldehyde, restriction enzyme digestion, and chromatin fragment ligation. The 3C assay is a PCR-based technology that determines the interaction frequencies between the potential interacting chromatin fragments by quantifying their ligation frequencies [10], [12], [18], [19]. In this study, there were eight *Hind* III-digested DNA fragments from the 3C assay including the bait CT6, which were aligned using the UCSC Genome Browser to determine their position on the *kcnq5* locus (Figure 2A). These fragments were named CTCF1 through CTCF8. CTCF1, CTCF5, and CTCF8 corresponded to the three 4C bait interaction partners No. 225, 319, and 202, respectively. The CTCF2, CTCF3, CTCF4, and CTCF6 fragments encompassed the potential CTCF binding sites identified using the UCSC Genome Browser. The CTCF7 fragment from 3C assay encompassed the highly conserved 4C bait CT6, while the CTCF2 fragment contained a CTCF binding site downstream of TSS and a proximal promoter region that was 1 kb upstream of the TSS. All 3C primers were designed to correspond to the sequences near the downstream sticky ends of the 3C fragments that resulted from *Hind* III digestion, which were named p1 to p8, respectively, according to their positions on the gene locus (Figure 2A and Table S2). To validate that these sites could be enriched by anti-CTCF chromatin immunoprecipitation (ChIP) or by other means of ChIP and formaldehyde-assisted isolation of regulatory elements (FIARE), the primers for the ChIP assays were designed to span the potential CTCF binding sites. In addition, one control site matched the promoter region located 1 kb upstream of the TSS (Table S3).

### The distribution of CTCF binding sites on the *Kcnq5* locus is cell-type specific

The human genome has approximately thirty thousand CTCF binding sites, but their distribution varies among different cell lines [13], [20]–[24]. As such, the distribution of cell-type specific CTCF binding sites on the *kcnq5* locus might determine the cell-type specific spatial organization mediated by CTCF. By performing qPCR analysis, the examined sites were efficiently enriched with anti-CTCF ChIP, except for the proximal promoter region (Figure 2B). The qPCR results were normalized to the same input template and showed various enrichment efficiencies at each of the examined sites. The interacting partners CTCF1, CTCF5, and CTCF8 identified in the 4C assay were all efficiently enriched, suggesting that they these three novel CTCF binding sites are present in MCF-7 cells. In addition, the ChIP qPCR results showed that the other sites (CTCF2, CTCF3, CTCF4, CTCF6, and CTCF7) were also efficiently enriched, which was consistent with the results obtained with the ENCONDE data sets regarding the other cell types using the UCSC Genome Browser. The control site of the promoter region was not enriched by anti-CTCF ChIP, which indicated that CTCF was absent at the *kcnq5* promoter region.

In order to test the cell-type specific organization of the gene locus, we also performed an anti-CTCF ChIP analysis of Jurkat cells to examine the cell type-specific distribution of CTCF binding sites on the *Kcnq5* gene locus. The ChIP qPCR results showed the cell type-specific enrichment (Figure 2C). In contrast, the CTCF2, CTCF3, CTCF4, and CTCF6 fragments as well as the promoter region were poorly enriched. These findings suggested that the enrichment efficiencies of the tested sites, with the exception of CTCF1, were lower in Jurkat cells compared to MCF-7 cells.

**Figure 3:**
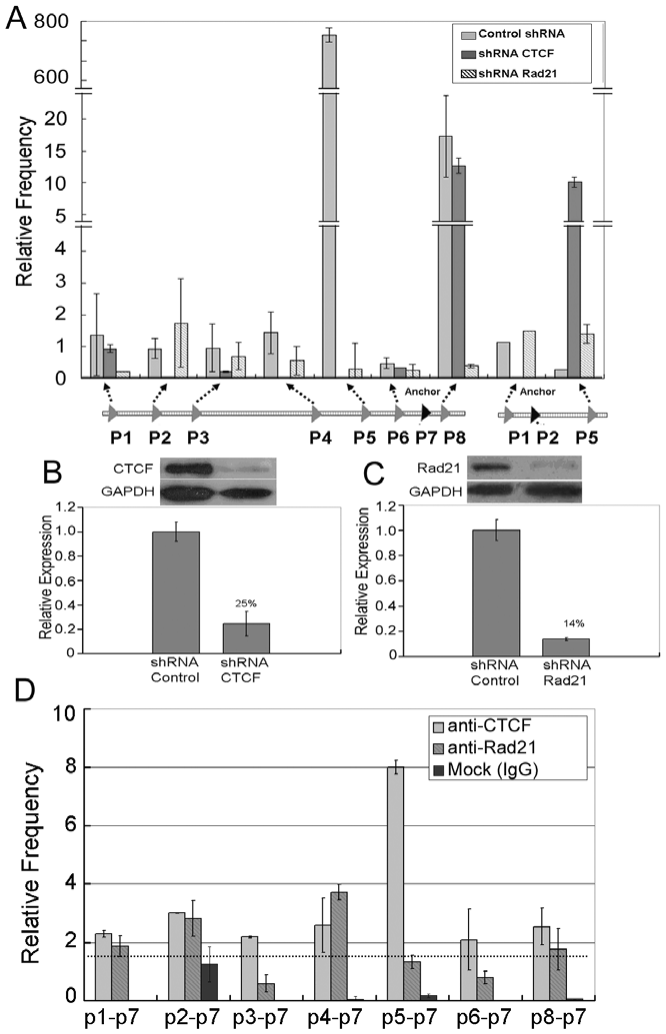
CTCF and Rad21 mediate the spatial organization of the *kcnq5* gene locus. (A) 3C quantitative PCR assay determined the interaction frequencies between the chromatin fragments of the *kcnq5* gene locus in MCF-7 cells with CTCF and Rad21 repression, respectively. The amplification efficiencies of the independent PCR reactions were normalized with respect to the control template. Error bars represent mean ± s.d. (n = 3). (B) Western blot (panel up) and reverse transcription quantitative PCR (RT-qPCR) (panel down) analyses of the CTCF expression level in MCF-7 cells after CTCF knockdown by CTCF shRNA. (C) Western blot (panel up) and RT-qPCR (panel down) analyses of the Rad21 expression level in MCF-7 cells after Rad21 knockdown by Rad21 shRNA. Transfection of MCF-7 cells with non-target shRNA was performed as a control. The relative transcription levels in the cells were normalized with respect to the endogenous control GAPDH. Error bars represent mean ± s.d. (n = 3). (D) Anti-CTCF and Rad21 ChIP-loop assays were performed with MCF-7 cells. The amplification efficiencies of the independent PCR reactions were normalized with the respect to the control template. Error bars represent mean ± s.d. (n = 3).

Several previous studies have suggested that a functional relationship exists between CTCF and Cohesin. Cohesin has four subunits, including Smc1, Smc3, SCC1 (MDC1 and RAD21), and SCC3 [23]–[25]. In order to determine whether Cohesin assists CTCF in mediating the spatial organization of the *kcnq5* gene locus, an anti-Rad21 ChIP assay was performed. However, the qPCR results from the ChIP assay revealed that very few sites were shared by both CTCF and Cohesin in the two cell lines (Figure 3B and C).

### The cell-type specific spatial organization of the *kcnq5* gene locus

The interaction frequencies of the 3C fragments were determined using the real-time qPCR assay with a SYBR green readout [10], [12], [18], [19]. The qPCR amplification efficiencies for the different primers pairs were normalized with respect to the control template. First, the 3C constant primer, p7, was anchored at the CTCF7 fragment, on which the 4C bait CT6 was located. The 3C assay determined the interaction frequencies between CTCF7 (p7) and other fragments (p1–p6 and p8). We then chose p2 as the 3C constant primer, which was anchored to the fragment of CTCF2 encompassing the proximal promoter region that was 1 kb upstream of TSS, in order to detect the interaction frequencies of p2 with p5 and p8.

The interactions occurring at the *kcnq5* gene locus in MCF-7 cells were determined using the 3C qPCR assay (Figure 2D). The results showed that the interaction frequencies of different pairs of 3C fragments were very distinct when normalized to the control template. We found that the relative interaction frequency between p7 and p5 was higher than the others by more than 100-fold, suggesting that the CTCF5 locus was spatially close to the CTCF7 locus even though there was a linear distance of more than 100 kb between them. The 3C qPCR assay results strongly indicated that most of the 3C fragment interactions were long-range interactions and had a linear distance of more than 100 kb from the CTCF7 (p7). In particular, the linear distance between CTCF7 (p7) and CTCF1 (p1) was more than 600 kb. Although the interaction frequency between CTCF8 (p8) and CTCF7 (p7) was also higher, the interaction was a type of short-range interaction with a linear distance of only 14 kb. Therefore, the linear proximity effect on the interaction frequency was greater than the others. Interactions between p2 and p1 as well as p5 were very low. Based on these data, the spatial organization of the *kcnq5* locus was confirmed by the 3C assay, which may be mediated by CTCF.

We also analyzed the spatial organization of the *kcnq5* gene locus in Jurkat cells using the 3C assay to determine the cell-type specific spatial organization of the gene locus. Interestingly, the interaction frequencies of the *kcnq5* gene locus in Jurkat cells were clearly distinguishable from MCF-7 cells normalized with respect to the same control template (Figure 2D). The most significant differences in interaction frequencies occurred between CTCF7 (p7) and CTCF8 (p8) as well as CTCF7 (p7) and CTCF5 (p5), while the highest interaction frequency occurred between p7 and p8 in Jurkat cells at a relative frequency that was greater than 100-fold compared to the others interactions. The interaction frequencies in Jurkat cells were generally lower following normalization with the same control template conditions compared to MCF-7 cells, except for the interaction frequency between p7 and p8. Although the interaction frequency between p7 and p8 was very high in Jurkat cells, the linear proximity effect on the interaction frequency was greater than the others with a linear distance of 14 kb. Therefore, the 3C assay confirmed that the spatial organization of the *kcnq5* gene locus was cell-type specific.

By comparing the CTCF binding ability with the interaction frequencies of the *kcnq5* locus mediated by CTCF in the different cell lines, we found that the cell-type specific distribution of CTCF binding sites in the *kcnq5* locus determined the CTCF-mediated cell-type specific organization of the locus.

### The cell-type specific higher-order chromatin structure of the *kcnq5* gene locus mediated by CTCF

In order to verify that the interactions occurring at the *kcnq5* gene locus were mediated by CTCF, we knocked down CTCF expression with RNAi in MCF-7 cells and then analyzed the cells using the 3C assay. As shown in Figure 3A, most of the interaction frequencies between these chromatin loops sharply decreased or even disappeared upon CTCF knockdown. The interaction frequencies of CTCF7 (p7) with CTCF1 (p1), CTCF3 (p3), CTCF6 (p6), and CTCF8 (p8) were greatly diminished, while interaction frequencies with CTCF2 (p2), CTCF4 (p4), and CTCF5 (p5) were almost completely abolished. The 3C assay results indicated that the spatial organization of the *kcnq5* gene locus was CTCF dependent and CTCF may be a master organizer of the global spatial organization of the *kcnq5* gene locus. Reverse transcriptional quantitative PCR (RT-qPCR) was used to analyze the expression level of CTCF in MCF-7 cells, and the results confirmed a high efficiency of CTCF knockdown. The transcription level was only 25.4% of that in the shRNA control cells, which was also confirmed by western blot analysis (Figure 3B).

We also knocked down Rad21 in MCF-7 cells, which is a subunit of Cohesin, using RNAi to test the role of Cohesin in spatial organization. Quantitative PCR assay results showed that Rad21 shRNA efficiently knocked down gene expression by 85.4% compared to the control cells, which was also confirmed by western blot analysis (Figure 3C). The effect of the repression Rad21 on *kcnq5* gene locus spatial organization was then characterized using the 3C assay. We found that when Rad21 was knocked down, the interaction frequencies between p7 and other sites also sharply decreased (Figure 3A), suggesting that Cohesin participated in the spatial organization of this gene locus. However, compared to the CTCF knockdown experiment, the influence of Rad21 knockdown was weaker. Therefore, these results support the hypothesis that CTCF mediates a network of long-range chromatin loop interactions at the *kcnq5* gene locus that involves Cohesin. In addition, the results from the ChIP-loop assay further support this conclusion.

ChIP is a powerful technique for studying genome wide protein-DNA interactions, while 3C and 3C-derived methods can detect long-range chromatin interactions. The ChIP-loop or ChIP-3C assays combined with ChIP and 3C can aid in our understanding of chromosome interactions in three dimensional spaces [26]–[28]. To further confirm the molecular mechanism behind the spatial organization of the *kcnq5* gene locus, we performed ChIP-loop assays with MCF-7 cells. The interaction frequencies between chromatin loops of the *kcnq5* locus were determined by quantitative PCR using anti-CTCF ChIP-loop assay (Figure 3D). These assays confirmed that the chromatin loop interactions occurred at the global gene locus and that long-range interactions were mediated by CTCF. These data further supported the hypothesis that CTCF plays a master role in the spatial organization process of the *kcnq5* gene locus. Similarly, the results of the anti-Rad21 ChIP-loop assay also showed that Cohesin participated in the partial chromatin loop interaction, which implied that Cohesin assists in the spatial organization of the *kcnq5* gene locus (Figure 3D).

### The cell-type specific higher-order chromatin structure of the *kcnq5* gene locus regulates local gene expressions

**Figure 4:**
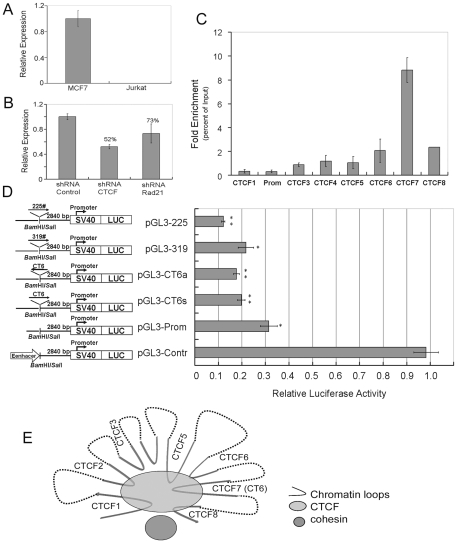
The dynamic spatial organization of *Kcnq5* locus and local gene regulation. (A) Reverse transcriptional quantitative PCR (RT-qPCR) analysis of KCNQ5 expression levels in MCF-7 and Jurkat cells. (B) RT-qPCR analysis of KCNQ5 relative transcription levels in MCF-7 cells transfected with CTCF shRNA, Rad21 shRNA, or shRNA control. The relative transcription levels in the cells were normalized with respect to the endogenous control GAPDH. Error bars represent mean ± s.d. (n = 3). (C) The quantitative PCR of the FAIRE assay with MCF-7 cells. The independent quantitative PCR amplification efficiency was normalized with the input template. Error bars represent mean ± s.d. (n = 3). (D) The *cis* regulatory element assays were performed in MCF-7 cells using the Luciferase Reporter System (Promega). Three DNA fragments of No. 225 (sense), No. 319 (sense), and CT6 (sense and antisense) were directly cloned into the *Bam* HI/*Sal* I site of the pGL3-promoter. The property of each target chromatin fragment was determined by the relative activity of the luciferase reporter. The values of the fluorescence units of firefly luciferase activities were normalized with Renilla luciferase activities (Firefly/Renilla). The values represent the average of independent quadruple repetition experiments and the error bars represent the standard deviation (n = 4); *: *P*<0.05; **: *P*<0.01. (E) A schematic representation of dynamic spatial organization at the *Kcnq5* locus. A schematic model representation of the CTCF-mediated dynamic spatial organization of the *kcnq5* locus, which regulates the local gene activity. CTCF mediates the contact of the repressive chromatin fragments within the inner gene locus and confines them within the hub with the assistance of cohesin.

KCNQ5 is a member of the KCNQ potassium channel gene family; however little is known about its regulation [14]–[16]. Our reverse transcriptional quantitative PCR (RT-qPCR) analysis revealed that KCNQ5 was also expressed at a high level in MCF-7 cells, but was repressed in Jurkat cells (Figure 4A). Therefore, we analyzed these two cell lines using the 3C assay to determine the cell-type specific spatial organization of the *kcnq5* locus that might regulate local gene expression. Results from the two cell lines confirmed our hypothesis that the spatial organization of the *kcnq5* gene locus was cell type-specific (Figure 4). By comparing the gene activity and gene locus organization in different cell lines, we hypothesized that the cell type-specific spatial organization of the *kcnq5* gene locus may functionally regulate the local gene activity. We further explored this hypothesis by knocking down the expression of CTCF and Rad21 in MCF-7 cells.

The 3C qPCR assay results showed that the interaction frequencies between chromatin loops that occurred in the *kcnq5* locus were greatly decreased after CTCF or Rad21 knockdown. In addition, the local gene expression was also repressed after CTCF or Rad21 knockdown. Reverse transcriptional qPCR assay results showed that KCNQ5 expression was down-regulated after CTCF and Rad21 knockdown in MCF-7 cells (Figure 4B). Moreover, CTCF repression influenced KCNQ5 expression more strongly than Rad21 repression with the 52% transcription levels of that in the control cells (Figure 4B). Taken together, these functional analyses supported the hypothesis that CTCF plays an important role in the spatial organization of the *kcnq5* gene locus and subsequent gene regulation.

### CTCF mediates the repressive *cis* regulatory elements to contact at the *Kcnq5* locus

Formaldehyde-assisted isolation of regulatory elements (FAIRE) is a convincing method used to isolate active regulatory elements genome wide. Active regulatory elements can escape the confines of the nucleosome and loop out, and these nucleosome-released DNA fragments are traditionally hypersensitive to DNase I and can be detected by FAIRE [29], [30]. Using FAIRE, we examined the enrichment efficiency of the sites detected at the *kcnq5* gene locus and found that they were distinct in MCF-7 cells (Figure 4C). The 4C bait CT6 (CTCF7) had particularly high enrichment, which was consistent with the previous report that CT6 is a ubiquitous DNase I HS site that overlaps with CTCF binding sites [13]. We then chose CT6, No. 225 (CTCF1), and No. 319 (CTCF5) as representative DNA fragments for *cis* regulatory element assays using the Luciferase Reporter System. In order to corroborate the possibility of a directional effect by the regulatory elements, we directly cloned the sense and antisense CT6 and inserted them upstream of the promoter, respectively. Using the Dual-Luciferase Reporter Assay (Promega), the relative Firefly luciferase activities of each independent experiment were normalized with respect to Renilla luciferase activity (Firefly/Renilla) (Figure 4D). Compared to the native pGL3-promoter control, all constructs showed that the reporters were significantly repressed. These results indicated that the tested chromatin fragments were repressive regulatory elements.

## Discussion

The dynamic interactions of chromatin have been implicated in various aspects of genes function and regulation, but the molecular mechanisms responsible for these interactions are still poorly understood. CTCF is a multi-functional factor that acts as a master organizer in dynamic nuclei architecture [9], [28], [31]–[33]. Here, we characterized the CTCF-mediated dynamic spatial organization of the *kcnq5* gene locus in detail to present a perspective of cell type-specific intra-chromosomal interaction networks that are established and maintained by both *cis*-elements and *trans*-factors.

CTCF binding ability is DNA methylation sensitive, which may influence CTCF disposition on the *kcnq5* gene locus and further influence gene locus organization [34], [35]. Therefore, we analyzed DNA methylation distribution on the *kcnq5* gene locus in the five cell lines (GM12878, H1-hESC, K562, HeLa-S3, and HepG2) that were previously reported by ENCODE using the UCSC Genome Browser. The analysis results showed that there was a similar distribution of DNA methylation at the *kcnq5* locus in all five cell lines, with DNA methylation occurring most frequently at the promoter region (Figure S1). No DNA methylation was detected at the other sites that we examined by anti-CTCF ChIP, which suggested that DNA methylation may not influence the CTCF binding site distribution on the *kcnq5* locus in MCF-7 cells.

On the other hand, CTCF binding to DNA and its occupancy were also influenced by Cohesin due to a functional relationship between the two proteins. Cohesin can influence CTCF binding stability and consequently the chromatin loop interaction mediated by CTCF [23], [24]. The results of the 3C assay in cells with Rad21 knocked down showed that Cohesin participated in the spatial organization of the *kcnq5* gene locus. The repression of Rad21 had the effect on the interaction frequencies between these chromatin loops (Figure 3A and C). However, the ChIP qPCR assay results showed that most of the analyzed sites were not efficiently enriched with anti-Rad21 ChIP of the MCF-7 cells (Figure 2B and C). These results suggested that Cohesin may not directly bind the chromatin fragments and mediate their interaction. Therefore, Cohesin could indirectly participate in the spatial localization of the gene locus by assisting CTCF disposition on the locus.

In this study, the dynamic spatial organization of the *kcnq5* locus in MCF-7 cells was characterized in detail. Functional analysis showed that the chromatin fragments of No. 225, 319, and CT6 were repressing regulatory elements in MCF-7 cells. The interactions between these chromatin fragments was mediated by CTCF and may encompass a tight hub (Figure 4E). The highest interaction frequency occurred between chromatin fragments of p7 (CT6) and p5 (319), suggesting that they are spatially closer in the center of the compartment. In contrast, the other interaction partners might be located at the periphery. The serials of repressing regulatory elements within the *kcnq5* gene locus were confined by CTCF.

The insulation function of CTCF in the global organization of nuclear architecture is predominantly due to the inhibition of communication between the enhancer and promoter, and the domain barrier which prevents the repressive chromatin domain to spread [5]–[9]. CTCF can functionally repress local gene activity by blocking the communication between the enhancer and promoter. Our results showed that CTCF mediated the contact of the repressive chromatin fragments within the gene locus and confine them within the hub. This global locus organization resulted in the up-regulation of local gene activity. When CTCF and Rad21 were repressed in MCF-7 cells, the interaction frequencies between the chromatin loops were sharply decreased or even disappeared, implying that the spatial organization status could not be maintained in the absence of these proteins (Figure 3A). In addition, functional analysis showed that the local gene was active in MCF-7 cells but was repressed when CTCF and Rad21 expression was knockdown. Therefore, the proposed model describes the dynamic process of the *kcnq5* gene locus, which can regulate local gene expression.

## Materials and Methods

### Cell culture and cross-linked cell nuclei preparation

MCF-7 cells were cultured in Dulbecco's modified Eagle's medium (DMEM; GIBCOBL) supplemented with 4 g/l D-glucose, 1 mM sodium pyruvate and 10% fetal calf serum (FCS). Jurkat cells were cultured in RPMI 1640 (Hyclone) containing 10% FCS. All cells were cultured at 37°C in a humidified incubator (Thermo Forma) with 5% carbon dioxide (CO_2_).

MCF-7 cells were cultured in 75 cm dishes for 48–72 h until the cells had reached 85–90% of the confluency. Cells were then fixed in 1% formaldehyde for 10 min, and the formaldehyde was quenched with 125 mM glycine for an additional 5 min. The cells were then rinsed with ice-cold PBS twice and harvested in ice-cold PBS. The harvested cell pellets were resuspended and lysed in cell lysis buffer [10 mM Tris (pH 8.0), 10 mM NaCl, 0.2% NP-40 (vol/vol), and protease inhibitors (Roche)]. Cell nuclei were isolated by centrifugation at 500 g for 8 min at 4°C. This method was used to isolate cell nuclei for ChIP, FAIRE, 4C, and 3C analyses.

### ChIP assay

The ChIP procedures used in this study were similar to those previously described [20]. CTCF (Millipore, 07-729) and Rad21 (AbCam, ab992) anti-serum were used for immunoprecipitation. The antibody-protein-DNA complexes were precipitated using Protein A/G Plus-Agarose Immunoprecipitation Reagent (sc-2003, Santan Cruz Biotech). Beads were washed 3 times with 1 ml low-stringency wash buffer [0.1% SDS (wt/vol), 1% Triton X-100 (vol/vol), 2 mM EDTA (pH 8.0), 150 mM NaCl, and 20 mM Tris-HCl (pH 8.0)] and once with 1 ml high-stringency wash buffer [0.1% SDS (wt/vol), 1% Triton X-100 (vol/vol), 2 mM EDTA (pH 8.0), 500 mM NaCl, and 20 mM Tris-HCl (pH 8.0)]. DNA samples were purified using the Qiaquick PCR Purification Kit (Qiagen). The ChIP Real-time Quantitative PCR assay was performed using iQ™5 Real-time PCR Detection Systems (Bio-Lab) with SYBR Premix Ex Taq™ (TaKaRa) as previously described [20].

### 4C assay

The 4C protocol was similar to that previously described [11], [17]. Briefly, cross-linked nuclei were digested with the restriction enzyme *Dpn* II. Nuclei (5×10^6^ cells) were resuspended in 270 µl of 1.1×*Dpn* II buffer containing with 0.1% SDS and incubated at 37°C for 1 h. Next, 30 µl 20% Triton X-100 (final concentration 2%) was added to quench the SDS and then the samples were incubated at 37°C for additional hour. Nuclei were digested with 300 U *Dpn* II (New England Biolabs, NEB) by incubating them overnight at 37°C. Before the ligation reaction, *Dpn* II was inactivated at 65°C for 25 min by the addition of 1% SDS. To enhance the susceptibility of DNA fragments in the cross-linked complexes to intra-molecular ligation and circularization, we minimized the DNA concentration in the ligation mixture and extended the ligation reaction time. Generally, the DNA sample concentration was 1–1.5 ng/µl, and the ligation time was approximately 16–20 h at 16°C with 1 U/µl T4 Ligase (New England Biolabs, NEB). After reverse cross-linking and overnight proteinase digestion at 65°C, the 4C DNA sample was precipitated at −70°C for 20 min with 0.3 M sodium acetate (pH 5.0) and 2.5 times the volume with ethanol. The pelleted DNA sample was resuspended with TE buffer (pH 8.0), and further purified using the Qiaquick PCR Purification Kit (Qiagen). The strategy for 4C analysis was based on nested reverse PCR. The second round PCR product was cloned into a T-vector and transformed into *E. coli*. Positive clones were identified by PCR and confirmed by sequencing.

### 3C assay

3C samples and control template were prepared using a modified procedure as previously described [10], [18], [19]. Cross-linked nuclei (5×10^6^ cells) were digested with 500 U of *Hind* III. The ligation concentration of digested genome DNA was lower than 3 ng/µl, and incubated with 1 U/µl T4 ligase (NEB) for 4 h at 16°C and followed by an incubation at room temperature for 30 min. The DNA samples were purified using the method described above for the 4C protocol. Control templates were prepared according to the PCR method description. The fragments were first amplified with the primers spanning *Hind* III sites downstream of the 3C fragments. After purification, the fragments were mixed at equimolar amounts and digested with *Hind* III. High concentration (100 ng/µl) ligations were then performed for 6–8 h at 16°C. The control template DNA sample was purified with the Qiaquick PCR Purification Kit (Qiagen).

The 3C qPCR assay primers were designed using Primers 3 software, which is available online for each specific application. Real-time Quantitative PCR was performed on iQ™5 Real-time PCR Detection Systems (Bio-Lab) using *SYBR Premix Ex Taq*™ (TaKaRa). Amplification efficiencies for all primers were normalized with respect to the control template. The linear range of amplification was determined for the experimental and control 3C templates with serial dilutions according to the start concentration. The amount of 3C template for each sample was normalized to equal the anchor 3C fragment amplification efficiency. The qPCR procedure (94°C for 30 s followed by 40 cycles of 94°C for 5 s, 59°C for 10 s, and 72°C for 10 s) was performed according to the manufacturer's instructions.

### ChIP-loop assay

The ChIP-loop assay followed the procedure that combined ChIP with 3C as previously described with some minor modifications [26], [27]. Briefly, after digesting the DNA with *Hind* III (NEB) for the 3C step, the cross-linked chromatin complexes were enriched with anti-CTCF or Rad21 ChIP. The immunoprecipitation complexes were resuspended in 200 µl of 1× T4 ligase buffer with 400 U of T4 ligase (NEB). DNA samples were purified with phenol/chloroform extraction and ethanol precipitation. Ligation efficiencies were determined using the qPCR assay as described above for the 3C assay.

### RNA isolation and genes expression analysis

Total RNA was isolated from MCF-7 or Jurkat cells with TRIzol reagent (Invitrogen) and purified using RNeasy mini kits (Qiagen). The cDNA was generated from 4 µg of total RNA using SuperScript™ III Reverse Transcriptase (Invitrogen) according to the manufacturer's instructions. Real-time assays were performed using iQ™5 Real-time PCR Detection Systems (Bio-Lab) with *SYBR Premix Ex Taq*™ (TaKaRa). Endogenous GAPDH levels were used to compare the gene amplification efficiencies of various samples. The RT-qPCR primers were designed online using Primers 3 software (Table S4).

### CTCF and Rad21 RNA interference

CTCF and Rad21 RNA interference was performed according to the siRNA strategy designed by Thermo Scientific Dharmacon (ON-TARGET*plus* siRNA Reagents, Thermo Scientific Dharmacon). The reference genes were as follows: human CTCF, NM_006565 and human RAD21, NM_006265. ON-TARGETplus non-targeting siRNA was performed as a control. The siRNA transfection of MCF-7 cells was performed in 6-well plates using the optimized *DharmaFECT* 1, which was recommended by the manufacturer. The cells were harvested 40 h after transfection.

### Formaldehyde-assisted isolation of regulatory elements (FAIRE) assay

The FAIRE assays were performed as previously described [29], [30]. Quantitative PCR assays were performed as described above for the ChIP assay.

### Luciferase reporter assay

DNA fragments from MCF-7 genomic DNA were amplified by PCR and cloned into a pGL3-promoter vector (Promega). The resulting plasmids, the native pGL3-promoter, or pGL3-control (200 ng) were transfected into MCF-7 cells using Lipofectamine™ 2000 (Invitrogen). The pRL-SV40 (Promega) renilla luciferase plasmid (20 ng) was also co-transfected according to the manufacturer's recommendations in 96-well plates. Each experiment was independently replicated in quadruplicate. Forty hours post-transfection, the luciferase activities of firefly and renilla were measured using the Dual-Luciferase Reporter Assay kit (Promega) according to the manufacturer's instructions. Fluorescence units were measured with the Precisely Envision-2103 Multilabel Reader luminometer (Perkin Elmer). The data were presented and normalized with respect to renilla luciferase activities (firefly/renilla) and analyzed by a Student's t-test. A *P* value<0.05 was considered statistically significant.

## Supporting Information

Figure S1 **The DNA Methylation distribution on** ***Kcnq5*** **locus.** The DNA methylation distribution on *Kcnq5* locus was analyzed with the data sets of ENCODE about five different cell lines using UCSC Genome Browser.
(DOC)

Table S1 **4C nested reverse PCR primers**
(DOC)

Table S2 **3C quantitative PCR primers**
(DOC)

Table S3 **ChIP quantitative PCR primers**
(DOC)

Table S4 **Gene transcriptional level quantitative PCR primers**
(DOC)
