# Correction: CTCF Mediates the Cell-Type Specific Spatial Organization of the *Kcnq5* Locus and the Local Gene Regulation

**DOI:** 10.1371/annotation/c6d76c92-dc6c-4102-86c0-03b996376778

**Published:** 2012-03-22

**Authors:** Licheng Ren, Yang Wang, Minglei Shi, Xiaoning Wang, Zhong Yang, Zhihu Zhao

There is an error in the affiliations of the first author. The correct affiliations for Licheng Ren are 1 State Key Laboratory of Bioreactor Engineering, East China University of Science and Technology, Shanghai, China, 2 Beijing Institute of Biotechnology, Fengtai District, Beijing, China. 

