# Supplementary figures and images for: CTCF Mediates the Cell-Type Specific Spatial Organization of the Kcnq5 Locus and the Local Gene Regulation
Source: PLoS One. 2012 Feb 8;7(2):e31416. doi: 10.1371/journal.pone.0031416 (PMC3275579; doi:10.1371/journal.pone.0031416)

**Supplemental Figure 1**

**Figure S1**


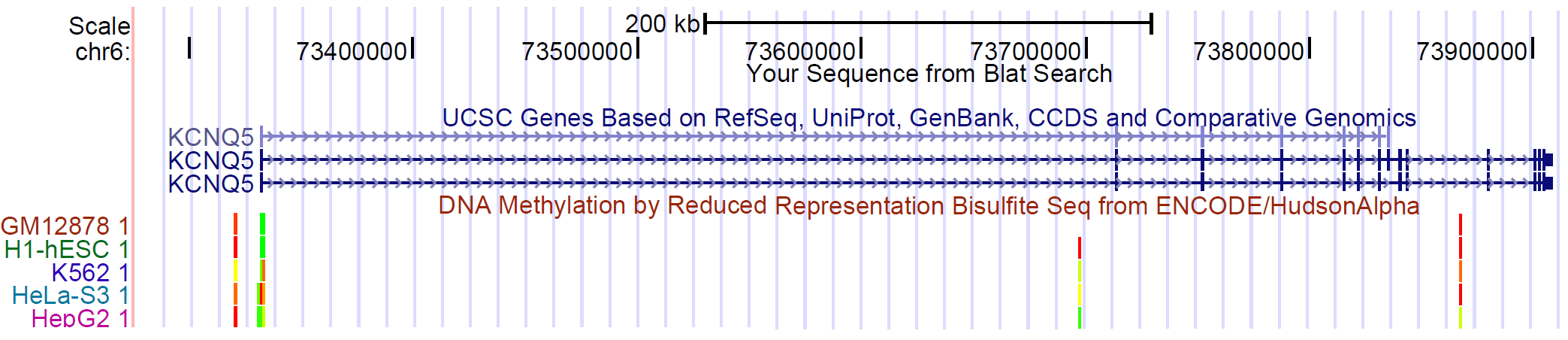

Supplement: Figure S1 — The DNA Methylation distribution on Kcnq5 locus. The DNA methylation distribution on Kcnq5 locus was analyzed with the data sets of ENCODE about five different cell lines using UCSC Genome Browser. (DOC) [file pone.0031416.s001.doc]
