# Supplementary material for: CTCF Mediates the Cell-Type Specific Spatial Organization of the Kcnq5 Locus and the Local Gene Regulation
Source: PLoS One. 2012 Feb 8;7(2):e31416. doi: 10.1371/journal.pone.0031416 (PMC3275579; doi:10.1371/journal.pone.0031416)
Supplement: Table S1 — 4C nested reverse PCR primers (DOC) [file pone.0031416.s002.doc]

**Supplemental Table 1**

**Table S1: 4C nested reverse PCR primers**

4Cfir-L 5’-GGAAAGTGCTCAGGGATTCTT-3’

4Cfir-R 5’-TCTCTTGCAGGGTGACAGTG-3’

4Csec-L 5’-GAGCATGGTCCTCAGCTGTAA-3’

4Csec-R 5’-TGTTGTTTAATTGTGCTGCTTG-3’
