# Supplementary material for: CTCF Mediates the Cell-Type Specific Spatial Organization of the Kcnq5 Locus and the Local Gene Regulation
Source: PLoS One. 2012 Feb 8;7(2):e31416. doi: 10.1371/journal.pone.0031416 (PMC3275579; doi:10.1371/journal.pone.0031416)
Supplement: Table S2 — 3C quantitative PCR primers (DOC) [file pone.0031416.s003.doc]

**Supplemental Table 2**

**Table S2: 3C quantitative PCR primers**

p1 5’-TGGAAGAAAGTGCCACATAGG-3’

p2 5’-AACACCAACACCAAGCAGTG-3’

p3 5’-TTCAATGCTGTTTCAAAAGGAC-3’

p4 5’-TCCTTTTCTTCCTTGACATTCC-3’

p5 5’-CGTTTCAAAGCCCAAGGTAG-3’

p6 5’-AAGAAAGATGGATGGTTTACGAA-3’

p7 5’-AAAGGTGCAGAAGTGAGCAT-3’

p8 5’-GCACAGTGCATGGCAAATAA-3’
