# Supplementary material for: CTCF Mediates the Cell-Type Specific Spatial Organization of the Kcnq5 Locus and the Local Gene Regulation
Source: PLoS One. 2012 Feb 8;7(2):e31416. doi: 10.1371/journal.pone.0031416 (PMC3275579; doi:10.1371/journal.pone.0031416)
Supplement: Table S3 — ChIP quantitative PCR primers (DOC) [file pone.0031416.s004.doc]

**Supplemental Table 3**

**Table S3: ChIP quantitative PCR primers**

Ctcf1-L 5’-GGGCAATGACCTCTTCCATA-3’

Ctcf1-R 5’-ACCCCACAGTTGCTTCAAAC-3’

Prom-L 5’-TGTCCACTGAACTGCTGAGG-3’

Prom-R 5’- GTGAAGTCAGCCTTGTGTCCT-3’

Ctcf2-L 5’-TTCCAAAGGTTCAAGGTTGG-3’

Ctcf2-R 5’-TAATTGCTGAGGCATTGCAG-3’

Ctcf3-L 5’-CCTCAGAGAGGCCTTGCATA-3’

Ctcf3-R 5’-AACCCCTCTGCATCTCTCAA-3’

Ctcf4-L 5’- TGGCAGTATGGAGAGGAACC-3’

Ctcf4-R 5’- CCACAGCAGCCACAGTAAGA -3’

Ctcf5-L 5’-TGAGCAACAAATTCACAAGTACG -3’

Ctcf5-R 5’-AGTAGAAGCGGTGGCAAGAG -3’

Ctcf6-L 5’- CAGCAAGGGGAAGACAGTTT -3’

Ctcf6-R 5’- CTCCCAGGCTAAACTCACCA -3’

Ctcf7-L 5'-ACCTGAGCCCTGTTCAGAGA -3’

Ctcf7-R 5'-TGGCAACTGAGCCATAACAA -3’

Ctcf8-L 5’-GTGGGGATACAGAGCGAAAC-3’

Ctcf8-R 5’-TCAATGGCAAAATGACTGAGA-3’
