# Supplementary material for: CTCF Mediates the Cell-Type Specific Spatial Organization of the Kcnq5 Locus and the Local Gene Regulation
Source: PLoS One. 2012 Feb 8;7(2):e31416. doi: 10.1371/journal.pone.0031416 (PMC3275579; doi:10.1371/journal.pone.0031416)
Supplement: Table S4 — Gene transcriptional level quantitative PCR primers (DOC) [file pone.0031416.s005.doc]

**Supplemental Table 4**

**Table S4: Gene transcriptional level quantitative PCR primers**

Gapdh-L 5’- GCCTTCCGTGTCCCCACTGC-3’

Gapdh-R 5’- CAATGCCAGCCCCAGCGTCA-3’

Kcnq5-L 5’-GGCATTCTTGGCTCAGGT-3’

Kcnq5-R 5’-AAGTGTGGCTTCCAGGTTG-3’

Ctcf -L1 5’-CCCACACCGGGGAGAAGCCT-3’

Ctcf -R1 5’-CGCCATCTGGGCCAGCACAA-3’

Rad21-L 5’-TCAGCAGATGCTTCATGGTC-3’

Rad21-R 5’-ACGGTTCTTCCTGTGTCAGC-3’
